# Letter to the Editor From Lin et al: “Testosterone Treatment, Weight Loss, and Health-Related Quality of Life and Psychosocial Function in Men: A 2-Year Randomized Controlled Trial”

**DOI:** 10.1210/clinem/dgaf066

**Published:** 2025-01-30

**Authors:** Kuo-Jen Lin, I Hung Shao, Yu-Hsiang Lin

**Affiliations:** Department of Urology, Chang Gung Memorial Hospital at Linkou, Taoyuan 333, Taiwan; School of Medicine, Chang Gung University, Taoyuan 333, Taiwan; Department of Urology, Chang Gung Memorial Hospital at Linkou, Taoyuan 333, Taiwan; School of Medicine, Chang Gung University, Taoyuan 333, Taiwan; Department of Urology, Chang Gung Memorial Hospital at Linkou, Taoyuan 333, Taiwan; School of Medicine, Chang Gung University, Taoyuan 333, Taiwan

To the Editor,

We read with great interest the recent study by Grossmann et al (1), which explored the effects of testosterone replacement therapy (TRT) on psychosocial outcomes within the framework of the T4DM trial. Their findings suggest that TRT did not provide significant psychosocial benefits compared to lifestyle interventions alone. This raises an intriguing point: could the use of a testosterone cutoff value of 400 ng/dL, as employed in the T4DM trial, have limited the ability to detect the psychological benefits of TRT?

The American Urological Association (AUA) defines testosterone deficiency as a level below 300 ng/dL, coupled with symptoms of low mood or energy (2). This threshold is rooted in its strong association with psychological well-being, particularly symptoms of low spirit. By adopting a higher cutoff value of 400 ng/dL, the T4DM trial likely recruited participants with relatively preserved psychosocial health. Consequently, this population may have been less likely to benefit from TRT in terms of psychological outcomes, as their baseline psychosocial function was likely closer to normal.

TRT has demonstrated multifaceted benefits in prior studies, including improvements in body composition, lipid profiles, glucose metabolism, and sexual parameters. Saad et al (3) comprehensively outlined the timelines for the onset and maximum effects of TRT in these domains, highlighting its broad therapeutic potential. Furthermore, the T4DM trial confirmed that TRT reduced the incidence of type 2 diabetes (T2DM) (4) and increased bone mineral density (5). However, extending the scope of TRT to psychosocial benefits in a population with preserved baseline function—as reflected by the 400 ng/dL threshold—may inherently limit the observable impact.

Beyond its metabolic effects, TRT has been linked to improvements in mitochondrial function, which plays a critical role in the pathogenesis of T2DM, cardiovascular disease, and neurodegenerative disorders (6, 7). Declining testosterone levels exacerbate mitochondrial dysfunction, a key driver of metabolic derangements associated with aging. This mechanistic link may partially explain the T2DM prevention effects of TRT observed in the T4DM trial (4). Exercise, another key intervention in the T4DM trial, also improves mitochondrial function (8). This dual benefit could rationalize the observed psychosocial improvements in participants undergoing lifestyle interventions alone, further reducing the detectable psychological impact of TRT in this trial.

The discrepancy between the AUA guideline's testosterone threshold of 300 ng/dL (2) and the T4DM trial's 400 ng/dL cutoff highlights an important methodological consideration. By recruiting participants with higher baseline testosterone levels, the T4DM trial may have inadvertently included individuals with less room for psychological improvement. This underscores the need for tailored cutoff values when evaluating domain-specific TRT outcomes, particularly for psychological health.

We propose that future studies stratify participants by baseline testosterone levels and assess outcomes using domain-specific thresholds. Such an approach would allow researchers to delineate the physiological and psychological domains where TRT is most beneficial and optimize treatment guidelines accordingly.
